# Editorial: Antifungal Pipeline: Build It Strong; Build It Better!

**DOI:** 10.3389/fcimb.2022.881272

**Published:** 2022-03-16

**Authors:** John R. Perfect, Damian J. Krysan, Maurizio Del Poeta, Anna M. Selmecki, Jessica C. S. Brown, Leah E. Cowen

**Affiliations:** ^1^ Division of Infectious Diseases, Department of Medicine, Duke University Medical Center, Durham, NC, United States; ^2^ Department of Molecular Genetics and Microbiology, Duke University Medical Center, Durham, NC, United States; ^3^ Department of Pediatrics and Microbiology, Carver College of Medicine, University of Iowa, Iowa City, IA, United States; ^4^ Department of Immunology, Carver College of Medicine, University of Iowa, Iowa City, IA, United States; ^5^ Department of Microbiology and Immunology and Division of Infectious Diseases, Stony Brook University, Stony Brook, NY, United States; ^6^ Veterans Administration Medical Center, Northport, NY, United States; ^7^ Department of Microbiology and Immunology, University of Minnesota Medical School, Minneapolis, MN, United States; ^8^ Department of Pathology, Division of Microbiology and Immunology, University of Utah, Salt Lake City, UT, United States; ^9^ Department of Molecular Genetics, University of Toronto, Toronto, ON, Canada

**Keywords:** antifungal, pipeline, invasive fungal infection (IFI), fungal disease, drug-resistance

The global burden of fungal infections remains unclear but there are estimates that they infect over a billion people with 150 million having serious fungal diseases and invasive fungal infections (IFIs) killing more than 1.5 million each year globally (Bongomin et al., 2017). More precise data has been reported that IFIs are associated with approximately 7.2 billion dollars in direct costs to the USA Healthcare System alone in 2017 (Benedict et al, 2019). Clearly, IFIs are common, deadly and costly.

Although there are reported to be more than 5 million fungal species worldwide, approximately 300 fungal species are implicated directly in human disease. Only a fraction (between 20-30 species) consistently produce disease. The mortality rates from IFIs remain high and drug- resistant molds (*i.e.* azole-resistant A*spergillus fumigatus*) or yeasts (*i.e. Candida auris*) have appeared globally and are spreading worldwide. Furthermore, the IFI onslaught has been accelerated by an enlarging global immunocompromised population resulting from persistent untreated HIV infections and ICU care as well as cancer and its new and old therapies. The “perfect storm” has arisen worldwide for the emergence of IFIs. Unfortunately, the medical community is armed with only four classes of antifungal agents for IFIs: the polyenes, azoles, echinocandins, and a pyrimidine analogue for fighting invasive fungal complications. In all these classes there has been success in IFI management but also occurrence of substantial defects and failures. Clearly, the numbers and outcomes for IFIs speak to the urgent demand for development of new effective antifungal agents.

However, there are a variety of important issues and potential roadblocks associated with the current state of antifungal development. First, the Gain Act and the Orphan Drug Act and Fast Track designation by USA FDA have provided a favorable climate for investment into antifungal agents. Unfortunately, antifungal drugs are unlikely to be “block busters” for pharmaceutical sales. Second, diagnosis has always been a challenge but new culture, serologies, genomic techniques, and biomarkers have improved the early diagnosis of IFIs but more improvements need to be made to facilitate the timely and accurate diagnosis of IFIs. Third, attributable mortality for IFIs with present antifungal agents remains too high (10-40% depending on the fungus in the best healthcare systems). Fourth, there needs to be an emphasis on antifungal agents that produce rapid fungicidal activity particularly in the context of reduced host immune functions. Furthermore, long-term treatment regimens interfere and complicate the treatment of underlying diseases; reduce compliance; increase drug toxicities; and amplify drug resistance pressures. Fifth, finding broad- spectrum antifungal agents are desirable because they allow less precise early diagnosis of a specific fungus and support their use in prevention strategies. However, the question remains how wide-spectrum and what fungi will be left out? Sixth, the concept of optimized combinations of antifungal agents may improve antifungal spectrum and potency by attacking multiple fungal targets. Although this strategy has been widely studied in the pre-clinical settings, its translation to patient care has lagged considerably due to challenges in both drug development and analysis. Seventh, the development of drug resistance in the fungal kingdom is present and measurable. There are no fungal drug-resistance transposons or plasmids that easily pass resistance between isolates, but all of our antifungal agents have been in use for at least twenty years; consequently, we are beginning to see the clinical impact of drug selection for resistance. Eighth, it is important to find new antifungal drugs with novel mechanism(s) of action that avoid targeting highly conserved eukaryotic proteins and processes because impacting human targets with cross-species target inhibition may add toxicity risks in patients that are already extremely fragile and vulnerable to organ damage. Simply put, new antifungal drugs must be safe. Ninth, the clinical development of these agents in a very sick population has significant logistical and financial challenges. Thus, strategies to improve patient recruitment to and retention in clinical studies are paramount (Perfect, 2017). Despite these challenges and the findings that approximately 80% of antifungal targets in the literature turn out to be false positives or possess little potential for development of target-based inhibitors (Pouliot and Jeanmart, 2016), the antifungal pipeline has substantially forged forward over the last decade. Over the last 1-2 years, we have received papers on the antifungal pipeline and these papers have described well the richness of the pipeline. Each paper has its own spin on this progress.

1. Wei et al. *Fingolimod Potentiates the Antifungal Activity of Amphotericin B*. This paper illustrates how we can improve a “potent gold standard” antifungal agent.
2. Alaalm et al. *Identification and Phenotypic Characterization of Hsp90 Phosphorylation Sites That Modulate Virulence Traits in the Major Human Fungal Pathogen Candida albicans.* The study illustrates a direct attack on the fungus to reduce its ability to produce disease. The strategy is to reduce the virulence potential of the fungus to help eliminate it from the host.
3. Gamal et al. *Ibrexafungerp, a Novel Oral Triterpenoid Antifungal in Development: Overview of Antifungal Activity Against Candida glabrata*. This study describes the newest class of antifungals to be approved by FDA for human use. It also focuses on a particularly troublesome yeast (*C. glabrata*) in the clinic with few good oral options for therapy today.
4. Gerlach et al. *ATI-2307 Exhibits Equivalent Antifungal Activity in Cryptococcus neoformans Clinical Isolates with High and Low Fluconazole IC 50.* This study describes a compound with a new unique mechanism of action focused on a mitochondrial target for rapid killing of an encapsulated yeast which still produces attributable mortality in 10-50% of patients with our present treatments.
5. Bapat and Nobile *Photodynamic Therapy Is Effective Against Candida auris Biofilms.* This paper has three messages: use a creative antifungal process that does not depend on a drug; attacking a drug-resistant yeast species; addresses biofilms as an antifungal problem structure for treatment.
6. McCarty and Pappas *Antifungal Pipeline.* This is a comprehensive and up-to-date review of the breadth of the new antifungal pipeline.
7. Seyedjavadi et al. *Characterization, Biological Activity, and Mechanism of Action of a Plant-Based Novel Antifungal Peptide, Cc-AFP1, Isolated From Carum carvi.* This paper brings in environmental sources for identification of antifungal compounds and re-examines peptides as an antifungal drug strategy.
8. Murphy and Bicanic *Drug Resistance and Novel Therapeutic Approaches in Invasive Candidiasis.* Candidiasis represents a common invasive mycoses across may clinical settings and the treatment failure rates remain high due to resistance mechanisms and host failures. This paper describes the new antifungal pipeline in relationship to this deadly yeast in modern medicine.

It is clear that multiple strategies are being leveraged to improve the antifungal pipeline. Of course, discovery of new antifungal compounds has been a primary focus of the pipeline but it is not limited to new chemical entities. Other strategies also include targeted delivery approaches, improving our existing antifungal agents, repurposing old drugs with other indications, host immune cell-targeted approaches and biological agents.

In sum, the arsenal needed for the war on IFIs must continue to be advanced on multiple fronts. IFIs are deadly, costly, and much too common for us to avoid in modern medicine. If anything, they are collateral damage from our success in areas such as organ transplantation and improved cancer treatments. We have made substantial progress since the initial use of amphotericin B deoxycholate in the 1960’s. The Antifungal Pipeline abounds with potential but faces challenges at all levels of its construction. IFIs remain in our present and future and the pipeline must be strong to eliminate or control the collateral damage of IFIs in immunosuppressed hosts. We cannot and will not accept the “status quo”.

## Author Contributions

All authors contributed equally to the writing and insights provided in this editorial and to the theme of this initiative on the Antifungal Pipeline” which it discusses.

## Conflict of Interest

The authors declare that the research was conducted in the absence of any commercial or financial relationships that could be construed as a potential conflict of interest.

## Publisher’s Note

All claims expressed in this article are solely those of the authors and do not necessarily represent those of their affiliated organizations, or those of the publisher, the editors and the reviewers. Any product that may be evaluated in this article, or claim that may be made by its manufacturer, is not guaranteed or endorsed by the publisher.
